# SGT1 is required in PcINF1/SRC2-1 induced pepper defense response by interacting with SRC2-1

**DOI:** 10.1038/srep21651

**Published:** 2016-02-22

**Authors:** Zhi-qin Liu, Yan-yan Liu, Lan-ping Shi, Sheng Yang, Lei Shen, Huan-xin Yu, Rong-zhang Wang, Jia-yu Wen, Qian Tang, Ansar Hussain, Muhammad Ifnan Khan, Jiong Hu, Cai-ling Liu, Yang-wen Zhang, Wei Cheng, Shui-lin He

**Affiliations:** 1 Ministry of Education Key Laboratory of Plant Genetic Improvement and Comprehensive Utilization, Fujian Agriculture and Forestry University, Fuzhou, 350002, China; 2 College of Crop Science, Fujian Agriculture and Forestry University, Fuzhou, 350002, China

## Abstract

PcINF1 was previously found to induce pepper defense response by interacting with SRC2-1, but the underlying mechanism remains uninvestigated. Herein, we describe the involvement of SGT1 in the PcINF1/SRC2-1-induced immunity. *SGT1* was observed to be up-regulated by *Phytophthora capsici* inoculation and synergistically transient overexpression of *PcINF1*/*SRC2-1* in pepper plants. *SGT1*-silencing compromised HR cell death, blocked H_2_O_2_ accumulation, and downregulated HR-associated and hormones-dependent marker genes’ expression triggered by *PcINF1*/*SRC2-1* co-overexpression. The interaction between SRC2-1 and SGT1 was found by the yeast two hybrid system and was further confirmed by bimolecular fluorescence complementation and co-immunoprecipitation analyses. The SGT1/SRC2-1 interaction was enhanced by transient overexpression of *PcINF1* and *Phytophthora capsici* inoculation, and *SGT1*-silencing attenuated PcINF1/SRC2-1 interaction. Additionally, by modulating subcellular localizations of SRC2-1, SGT1, and the interacting complex of SGT1/SRC2-1, it was revealed that exclusive nuclear targeting of the SGT1/SRC2-1 complex blocks immunity triggered by formation of SGT1/SRC2-1, and a translocation of the SGT1/SRC2-1 complex from the plasma membrane and cytoplasm to the nuclei upon the inoculation of *P. capsici*. Our data demonstrate that the SGT1/SRC2-1 interaction, and its nucleocytoplasmic partitioning, is involved in pepper’s immunity against *P. capsici*, thus providing a molecular link between Ca^2+^ signaling associated SRC2-1 and SGT1-mediated defense signaling.

Elicitins, conserved extacellular proteins in *Phytophthora* and *Pythium* spp., which might have evolved to the benefit of pathogens, trigger immunity in the host plants such as *Nicotiana benthamiana* due to the presence of ligands that have been evolved under the selective pressure. Unlike the well-characterized typical PAMPs and effectors, elicitins are rather narrowly conserved and transported into plant cells by receptor-mediated endocytosis^1^ possibly through a clathrin-mediated way^2^,^3^, thereby triggering hypersensitive reaction (HR) cell death in the host plants, which is a hallmark of effector-triggered immunity (ETI)^4^,^5^,^6^,^7^,^8^,^9^. These features suggest their roles as elicitors in the continuum between typical PAMPs and effectors. Upon treatment with elicitins, cellular responses including depolarization of the plasma membrane, potassium and chloride ion efflux, a large calcium ion influx, alkalization of the extracellular medium, and production of either reactive oxygen species or nitric oxide are activated and culminated in the HR and plant immunity^10^. These processes are believed to be initiated by the recognition and binding of elicitins to their corresponding receptors in plant plasma membranes. Although NgRLK1, a receptor like kinase, and NbLRK1, a lectin-like receptor kinase, both in Nicotiana, have been shown to interact with the elicitins capsicein and INF1, respectively^11^,^12^,^13^. However, the receptors of most elicitins have not been identified. PcINF1, a member of the elicitins from *Phytophthora capsici* exhibiting a high sequence homology with orthologs in other *Phytophthora* species, was found to trigger HR cell death in tobacco. Additionally, our previous study further found that PcINF1 triggers HR cell death and defense response in pepper and *Nicotiana benthamiana* plants by interacting with SRC2-1^14^ which was found to be induced by pathogen inoculation as well as by treatment with abiotic stresses. Noticeably, the SRC2-1 contains a conserved C2 domain, which was originally identified in Ca^2+^-dependent isoforms of protein kinase C and is present in most Ca^2+^-binding proteins involved in protein-protein interactions, binding of phospholipids, and targeting of proteins to the membrane in response to Ca^2+^ signalling^15^,^16^,^17^. Since there is no any kinase domain in SRC2-1 as in NgRLK1, NbLRK1 and other typical PRRs, and C2 domains have been implicated in protein-protein interactions^2^. SRC2-1 may form complexes with other proteins such as receptor-like kinases to elicit defense response in host plants. However, little is known about the components of these complexes, as well as the molecular events leading to the defense response triggered by PcINF1.

The highly conserved eukaryotic co-chaperone SGT1 (suppressor of the G2 allele of *skp1*) is an important protein component shared by PTI (PAMP-triggered immunity)^3^ and ETI (effector-triggered immunity)^18^,^19^,^20^,^21^,^22^. When involved in ETI, SGT1 mediates R proteins, such as Rps^4^, N^5^, Rpi-blb2^20^, Rx1^23^, R3a^24^, RPS4^25^, and MLA^26^, controlling their stability and activity as well as nucleocytoplasmic balance^22^,^27^. SGT1 also facilitates the recognition of effectors by R proteins^28^. However, some R proteins might function independently of SGT1^29^. SGT1 has also been found to participate in the regulation of plant PTI. For example, SGT1 is significantly induced by Xcc-derived flg22, a typical PAMP, as well as the flg22 domain-containing hook-associated protein (Fla) in citrus and in *Nicotiana benthamiana*^30^,^31^. SGT1 functions as an integral component in both ETI and PTI by interacting with other immunity-associated components such as RACK1, Rac1, RAR1, Rboh, HSP90, and HSP70^22^,^32^,^33^,^34^,^35^,^36^,^37^,^38^,^39^ to create multi-protein networks. Recently, it was found that SGT1 might act as a receptor for the type III effector AvrBsT^6^ by interacting with receptor-like cytoplasmic kinase1 in pepper. How SGT1 mediates the signaling of the network/s, however, it is not fully understood; other interacting partners of SGT1 may exist. Further identification and characterization of binding partners may help to elucidate the mechanisms of plant immunity.

In the present study, SGT1 was found to be essential in the defense response induced by PcINF1 by interacting with SRC2-1. Furthermore, the translocation of the SGT1/SRC2-1 complex from membrane and cytoplasm into nuclei was induced by pathogen inoculation and is required for their roles in immunity. Additionally, a functional association between the two proteins was observed through their synergistic response to pathogen inoculation.

## Results

### Transcriptional expression of *SGT1* in response to *Phytophthora capsici* inoculation and *PcINF1*/*SRC2-1* transient overexpression

**Figure 1:**
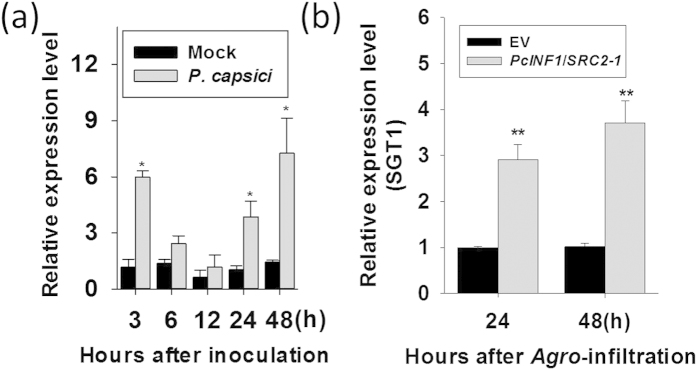
Transcript levels of *SGT1* in response to *Phytophthora capsici* infection and the transient overexpression of PcINF1/SRC2-1 in pepper leaves. (**a**) The *SGT1* transcript levels detected at different time points in pepper leaves inoculated with *P. capsici*. The 4-weeks-old pepper plants growing in the greenhouse were used for *P. capsici* infection. After *P. capsici* inoculation, the leaves next to the infected leaves of the pepper plants were harvested for RNA extraction at different time points. Pepper leaves treated with MgCl_2_ was used as a negative control (Mock). (**b**) The *SGT1* transcript levels in pepper leaves transiently overexpressing the PcINF1/SRC2-1 fusion protein. Agrobacterial cells harboring the construct containing the PcINF1/SRC2-1 fusion vector or the empty vector (EV) were infiltrated into the leaves of the healthy pepper plants. 24 h and 48 h after *Agro*-infiltration, the *Agro*-infiltrated pepper leaves were collected for total RNA extraction. (**a,b**) Experiments were repeated with at least four independent biological repetitions. Error bars indicate standard deviation (SD). Asterisks indicate a significant difference using a Fisher’s protected least significant difference (LSD) test. *P < 0.05; **P < 0.01.

*Phytophthora capsici* is a causal agent of phytophthora blight of pepper, one of the most important diseases in pepper production, and elicitins are conserved extracellular proteins in *P. capsici* and other *Phytophthora* and *Pythium* spp. triggering immunity in the host plants. Our previous study found that SRC2-1 from pepper plants perceive and interact with PcINF1 and trigger HR cell death and immunity^14^, but the underlying mechanism remains unknown. To isolate potential SRC2-1-interacting partners, a pepper cDNA library generated from *P. capsici*-inoculated pepper leaves was previously screened with SRC2-1 as a bait using GAL4-based yeast two-hybrid system, one positive clone acquired was identified to be *SGT1*. Since *SGT1* have been implicated in plant immunity^40^, the expression of *SGT1* was assayed by real-time PCR in *P. capsici*-inoculated pepper plants to test its potential role in immunity against *P. capsici* inoculation. The result showed that the expression of *SGT1* was enhanced compared to the mock treatment (Fig. 1a). Our previous study found that PcINF1, an elicitin from *P. capsici*, triggered HR cell death and defense response in pepper plants by interacting with SRC2-1^14^. To test if SGT1 is involved in the immunity mediated by PcINF1/SRC2-1, an *Agrobacterium*-mediated transient expression system was employed to characterize the effects of *PcINF1*/*SRC2-1* simultaneously transient overexpression on the expression of *SGT1*. Agrobacterial cells containing 35S::*PcINF1*/35S::*SRC2-1* fusion vector were infiltrated into pepper leaves, and the transcript level of *SGT1* was assayed by real-time RT-PCR (Fig. 1b). The result showed that the transcript levels of *PcINF1* and *SRC2-1* were both significantly increased in the pepper leaves transiently expressing *PcINF1* and *SRC2-1* (Supplementary Fig. S1), additionally, *SGT1* was also upregulated by the *PcINF1*/*SRC2-1* transient overexpression, indicating that SGT1 may be involved in pepper immunity mediated by PcINF1/SRC2-1.

### The Silencing of *SGT1* compromises the immunity triggered by PcINF1/SRC2-1

**Figure 2:**
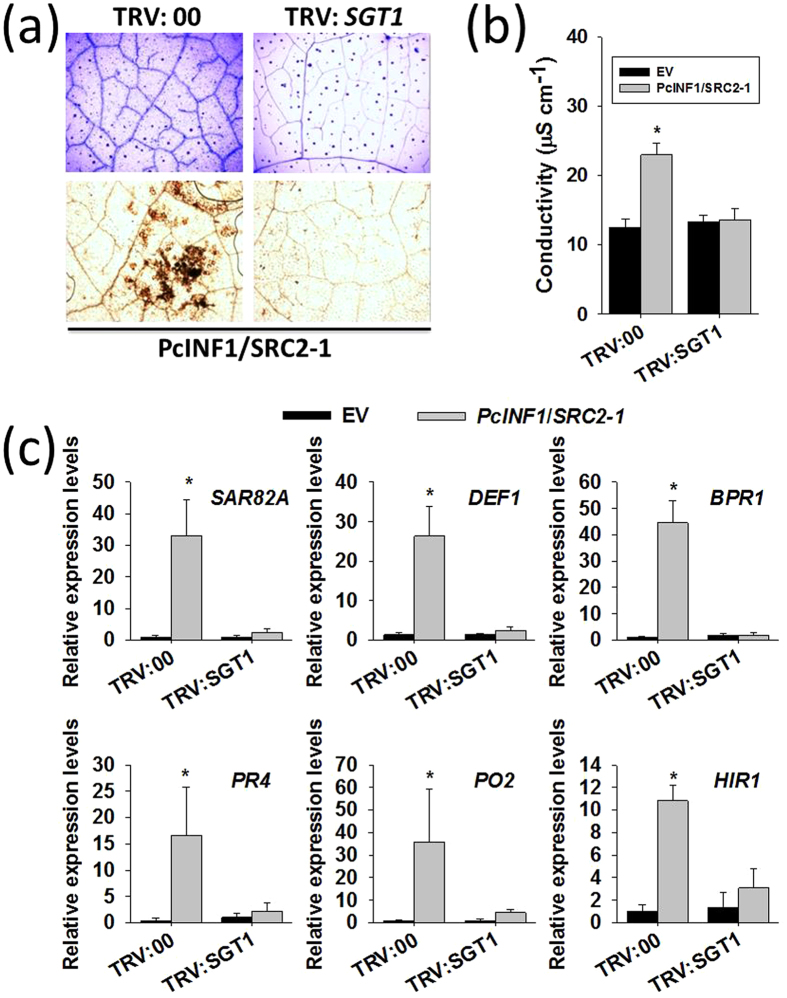
Silencing of *SGT1* restrained the defense response triggered by PcINF1/SRC2-1 complex in pepper plants. (**a**) Trypan blue and DAB staining of *SGT1*-silenced and unsilenced pepper leaves transiently expressing PcINF1/SRC2-1 vector. Trypan blue and DAB staining were performed 48 h after *Agro*-infiltration to detect the cell death and H_2_O_2_ content. (**b**) Electrolyte leakage measurement of *SGT1*-silenced pepper leaves transiently expressing PcINF1/SRC2-1. The transcript level of *SGT1* in pepper leaves was first silenced using the VIGS technology. The GV3101 cells harboring the constructs of PcINF1/SRC2-1 or empty vector were infiltrated into the leaves of *SGT1*-silenced and unsilenced pepper leaves. The leaves were collected 48 h after *Agro*-infiltration for the electrolyte leakage measurement. (**c**) Relative expression levels of defense-related marker genes in pepper leaves 48 h after *Agro*-infiltration with GV3101 cells harboring the PcINF1/SRC2-1 fusion vector, compared to empty vector, which was set to a relative expression level of “1”. *CaActin* and 18S ribosomal RNA were used to normalize the transcript level. (**b,c**) Experiments were repeated with at least four independent biological repetitions. Error bars indicate SD. Asterisks indicate a significant difference using an LSD test. *P < 0.05; **P < 0.01.

To further confirm the potential role of SGT1 in the defense response triggered by PcINF1/SRC2-1, the silencing of *SGT1* was performed by a VIGS (virus induced gene silence), which has been successfully used in Solanaceae, such as pepper, tomato, and tobacco^14^,^41^,^42^,^43^. To avoid the mistargeting of any potential orthologs of *SGT1*, fragment of 213 bps in length corresponding to a specific region of the 3′UTRs of *SGT1* was chosen to construct VIGS vectors (The specificity of the region was confirmed by BLASTN in pepper genome databases: http://passport.pepper.snu.ac.kr/?t=PGENOME and http://peppersequence.genomics.cn/page/species/index.jsp). The efficiency of *SGT1* silencing was examined by the real time RT-PCR and the result showed that the expression of *SGT1* was significantly decreased in the TRV:*SGT1* pepper plants (Supplementary Fig. S2). In *SGT1*-silenced pepper plants, the HR cell death manifested with the darker trypan blue staining triggered by the transient co-overexpression of *PcINF1*/*SRC2-1* significantly decreased compared to the control plants (Fig. 2a), and was consistent with an increased ion leakage in the *PcINF1*/*SRC2-1* co-overexpressed and *SGT1*-silenced pepper plants (Fig. 2b). In addition, the H_2_O_2_ accumulation triggered by the co-overexpression of *PcINF1*/*SRC2-1* manifested by the DAB staining (Fig. 2a), which is closely related to HR cell death by previous studies^40^,^44^, was also found to be compromised by *SGT1* silencing. Furthermore, the SA-dependent *SAR82A*, *BPR1* and *PR4*, JA-dependent *DEF1*, HR associated marker gene *HIR1* and defense-associated *PO2* (triggered by the *PcINF1*/*SRC2-1* co-overexpression) were also consistently compromised by the silencing of *SGT1* (Fig. 2c). All these results suggest that SGT1 is involved in pepper immunity mediated by PcINF1/SRC2-1.

### Simultaneously transient overexpression of *SGT1* and *SRC2-1* induced heightened cell death and defense responses in pepper leaves

**Figure 3:**
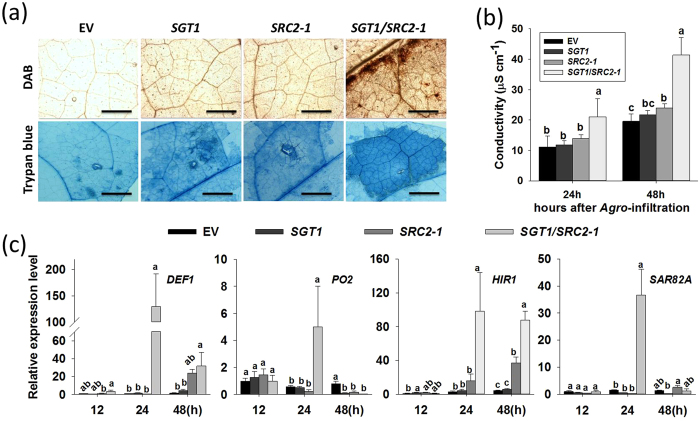
Transient co-expression of *SGT1* and *SRC2-1* induced cell death in pepper leaves. (**a**) The DAB and trypan blue staining of the *Agro*-infiltrated pepper leaves. The DAB and trypan blue staining assay were performed at 48 h after infiltration with GV3101 cells harboring the constructs containing *SGT1*, *SRC2-1*, *SGT1*/*SRC2-1*, and EV (empty vector), respectively. (**b**) Electrolyte leakage in *Agro*-infiltrated pepper leaves at 24 and 48 hours post infiltration. The pepper leaves was infiltrated with GV3101 cells harboring constructs containing *SGT1*, *SRC2-1*, *SGT1*/*SRC2-1*, and EV (empty vector) grown to an OD_595_ of 0.4, the infiltrated pepper leaves were collected at different time points for the electrolyte leakage measurement. (**c**) Quantitative real-time RT-PCR analysis of the transcript levels of stresses-associated marker genes in pepper leaves transiently expressing *SGT1* and *SRC2-1* at different time points after inoculation with *P. capsici*. Expression values are normalized by the expression level of *CaActin and* 18S ribosomal RNA. (**b,c**) Data represent mean ± SD from three independent biological repetitions. Different letters indicate statistically significant differences using an LSD test (*P* < 0.05).

Since both SRC2-1 and SGT1 are involved in the pepper defense response against *P. capsici* inoculation as well as the simultaneously transient overexpression of *PcINF1*/ *SRC2-1*^14^, it appears that these two proteins are functionally related. To test this possibility, the effect of the simultaneously transient co-overexpression of *SRC2-1* and *SGT1* on the defense response in pepper plants was assayed. Agrobacterial cells containing 35S::*SGT1* and 35S::*SRC2-1* were infiltrated into pepper leaves individually or simultaneously. Infiltrated pepper leaves were then harvested at either 48 HAI (for trypan blue staining, DAB staining, and conductivity measurements), or at 12, 24, and 48 HAI for total RNA isolation to detect the transcriptional expression of defense-associated marker genes. Our results showed that both SGT1 and *SRC2-1* were successfully overexpressed in the pepper leaves infiltrated with agrobacteria cells carrying 35S::*SGT1* and 35S::*SRC2-1* (Supplementary Fig. S3), besides, transient co-overexpression of *SGT1* and *SRC2-1* in pepper leaves induced significant HR-like cell death displayed by the trypan blue staining, the accumulation of H_2_O_2_, and the ion leakage measurement (Fig. 3a/b). This was coupled with significant up-regulation of HR-marker genes *HIR1*, SA-dependent *SAR82A*, JA-dependent *DEF1*, and active oxygen metabolism-associated *PO2*. Up-regulation of these genes occurred at 12, 24, or 48 HAI with maximum expression occurring at different time points for each gene (Fig. 3c). Additionally, the heightened cell death and the accumulation of H_2_O_2_, as well as the up-regulation of HR-, SA-, and JA-associated marker genes *DEF1*, *PO2*, *HIR1*, and *SAR82A*, were triggered by the simultaneous co-overexpression of *SGT1* and *SRC2-1*.

### Simultaneous silencing of *SGT1* and *SRC2-1* compromises immunity to a greater extent compared to single gene silencing in pepper plants against *P. capsici* inoculation

**Figure 4:**
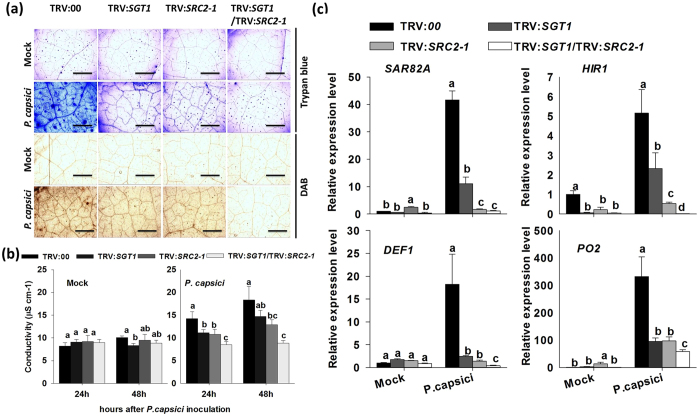
Silencing of *SGT1*, *SRC2-1*, and *SGT1*/*SRC2-1* compromises the hypersensitive cell death response in pepper leaves inoculated with *P. capsici*. (**a**) The trypan blue and DAB staining of *SGT1*-, *SRC2-1*-, and *SGT*1-/*SRC2-1*- silenced pepper leaves 24 h post inoculation with *P. capsici*. *SGT1* and *SRC2-1* were silenced in pepper plants using the virus-induced gene silencing technology. The *P. capsici* zoospores were dissolved in sterilized ddH_2_O and infiltrated into the leaves of the pepper plants with a syringe without needle. (**b**) Electrolyte leakage measurements of the VIGS and control pepper leaves collected at different time points after *P. capsici* inoculation. The *P. capsici* inoculated leaves of the VIGS and control pepper plants were harvested at 24 and 48 hpi for electrolyte leakage measurement. (**c**) Quantitative real-time RT-PCR analysis of the expression of stress-related marker genes in leaves of VIGS and control (TRV:*00*) pepper plants 24 h after inoculation with *P. capsici*. Expression values are normalized to the expression level of *CaActin* and 18S ribosomal RNA. (**b,c**) Data represents the mean ± SD from three independent biological repetitions. Different letters indicate statistically significant differences as analyzed by an LSD test (*P* < 0.05). Mock, treated with 10 mM MgCl_2_.

To characterize the functional relationship between *SGT1* and *SRC2-1* by loss of function by VIGS, GV3101 cells containing the constructs TRV:*SGT1* or TRV:*SRC2-1* were infiltrated into 4-leaf-age pepper plants individually or simultaneously. The silencing efficiency was examined by qPCR (Supplementary Fig. S2). The results show that *SGT1* and *SRC2-1* were successfully silenced in the corresponding pepper plants. The silencing of either *SGT1* or *SRC2-1* conferred decreased HR cell death and H_2_O_2_ accumulation, manifested by weaker trypan blue and DAB staining, and also a decrement on electrolyte leakage (Fig. 4a,b). Additionally, the down-regulation of HR cell death and SA- and JA-dependent immunity-associated marker genes was observed (Fig. 4c). However, simultaneous *SGT1* and *SRC2-1* silencing reduced cell death indicators and the expression of immunity-associated genes (including SA-dependent *SAR82A*, HR-marker genes *HIR1*, JA-dependent *DEF1*, and active oxygen metabolism-associated *PO2*) to a much lower level compared to that resulting from the individual silencing of either gene alone or the unsilenced control plants.

### Analysis of SGT1 and SRC2-1 inter-dependence in immunity induction using VIGS and transient overexpression

**Figure 5:**
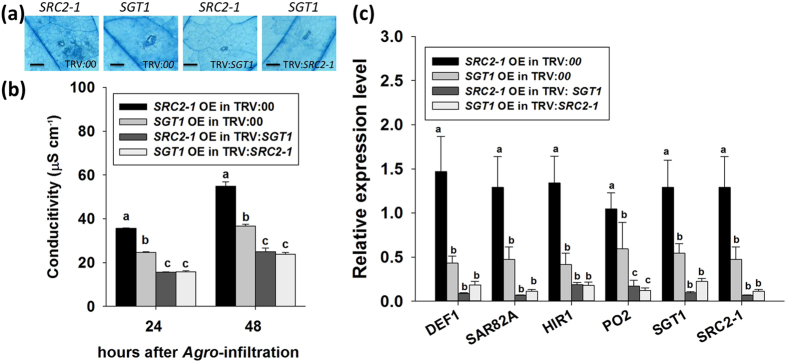
The transcript levels of stress-related marker genes in *SGT1*- and *SRC2-1*-silenced pepper leaves transiently expressing either *SRC2-1* or *SGT1*, respectively. (**a**) The trypan blue staining of VIGS pepper leaves transiently overexpressing *SGT1* or *SRC2-1*. GV3101 cells harboring the *SGT1* and *SRC2-1* overexpression vectors were infiltrated into the *SRC2-1*- and *SGT1*-silenced pepper leaves, respectively. The trypan blue staining assay was performed at 48 hpi. (**b**) Electrolyte leakage measurements of *SGT1-* or *SRC2-1* silenced pepper leaves 24 and 48 h post infiltration with GV3101 cells containing *SRC2-1* or *SGT1* overexpression vectors. (**c**) Quantitative real-time PCR analysis of the defense response associated marker genes in the VIGS and control pepper leaves transiently overexpressing either *SRC2-1* or *SGT1*. (**b,c**) Data represents the mean ± SD from three independent biological repetitions. Different letters indicate statistically significant differences as analyzed with an LSD test (*P* < 0.05).

To investigate whether SGT1 and SRC2-1 are interdependent on each other for their roles in pepper immunity, a combination of VIGS and transient overexpression was employed to investigate the function of each protein independently. The results show that in pepper plants with *SRC2-1* silenced, but with *SGT1* transiently overexpressed, the cell death intensity and electrolyte leakage iron were both lower than that in control plants transiently overexpressing *SGT1* (Fig. 5a,b). Additionally, the transcriptional expression of HR-associated *HIR1*, SA-dependent *SAR82A*, and JA-dependent *DEF1* were significantly lower than that in control plants transiently overexpressing *SGT1* (Fig. 5c). Similarly, the cell death intensity and the expression of these immunity-associated genes were also inhibited in the *SGT1-*silenced pepper plants transiently overexpressing *SRC2-1* when compared to the control plants overexpressing *SRC2-1* (Fig. 5).

### SGT1 interacts with SRC2-1

**Figure 6:**
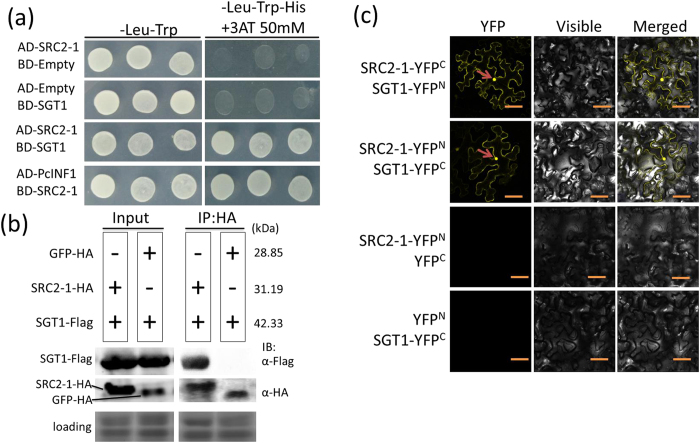
The interaction identification between SGT1 and SRC2-1 or PcINF1 by yeast two- hybrid, co-IP and BiFC. (**a**) The interaction confirmation between SGT1 and SRC2-1 by yeast two-hybrid. Yeast transformants expressing the corresponding serial vectors were plated on SD-Leu-Trp-His media containing 50 mM 3-amino-1, 2, 4-triazole (3AT), a competitive inhibitor of the His3p enzyme. The PcINF1/SRC2-1 interaction was used as positive control. (**b**) The SGT1/SRC2-1 interaction confirmation by co-IP. SRC2-1-HA/SGT1-Flag and GFP-HA/SGT1-Flag protein complexes co-expressed in *N. benthamiana* leaves (OD_595_ = 0.8). Anti-HA and anti-Flag antibodies were used to detect SRC2-1 (GFP) and SGT1, respectively. The interaction of GFP-HA and SGT1-Flag were used as a negative control. Coomassie brilliant blue staining of the membrane was to show the equal loading. IB: immunoblotting. (**c**) BiFC assay of the SGT1/SRC2-1 interaction in *N. benthamiana* leaves infiltrated with *Agrobacterium tumefaciens* (OD_595_ = 0.4). The fluorescent signals were detected using a confocal microscope 48 h after infiltration. Bars = 50 μm.

The above data suggests that SGT1 is involved in PcINF1/SRC2-1 mediated pepper immunity, and that SRC2-1 and SGT1 are functionally related with each other. Furthermore, it appears that SRC2-1 and SGT1 may be functionally related in a protein-protein interacting manner. To test this possibility, the interaction between the two proteins was assayed using the yeast two-hybrid (Y2H) system. The Y2H analysis showed that SRC2-1 interacts with SGT1 (Fig. 6a).

Co-IP assays were used to investigate SGT1/SRC2-1 binding. Co-IP was performed using the same transient co-expression system for leaves of *N. benthamiana*. This result suggests that SRC2-1 binds to SGT1 (Fig. 6b). The interaction of PcINF1 and SRC2-1 was used as positive control. The negative control experiment, which used GFP-HA and SGT1-Flag, showed no sign of interaction (Fig. 6b).

Additionally, SGT1/SRC2-1 interaction was further confirmed by BiFC analysis. The N- and C-terminal of yellow fluorescent protein (YFP) were fused to SGT1 and SRC2-1 to generate SGT1-YFP^N^ and SRC2-1-YFP^C^. The reciprocal constructs (SGT1-YFP^C^ and SRC2-1-YFP^N^) were also generated. All of the destination constructs were introduced into the *Agrobacterium* strain GV3101 for *Agrobacterium*-mediated transient overexpression in *N. benthamiana* leaves. 48 HAI, YFP signal in *N. benthamiana* leaves was detected using the leica laser confocal microscopy, with SRC2-1-YFP^N^/YFP^C^ and SGT1-YFP^C^/YFP^N^ being used as negative controls. Transient co-expression of SGT1-YFP^C^/SRC2-1-YFP^N^ and SGT1-YFP^N^/SRC2-1-YFP^C^ in *N. benthamiana* leaves resulted in the presence of yellow fluorescence in the membrane, cytoplasm, and nuclei, indicating that SGT1 interacts with SRC2 in the membrane, cytoplasm and the nucleus of the plant cells. The YFP signal was not detected in the negative control (Fig. 6c).

### The effect of *PcINF1* transient overexpression and *Phytophthora capsici* inoculation on SRC2-1/SGT1 interaction

**Figure 7:**
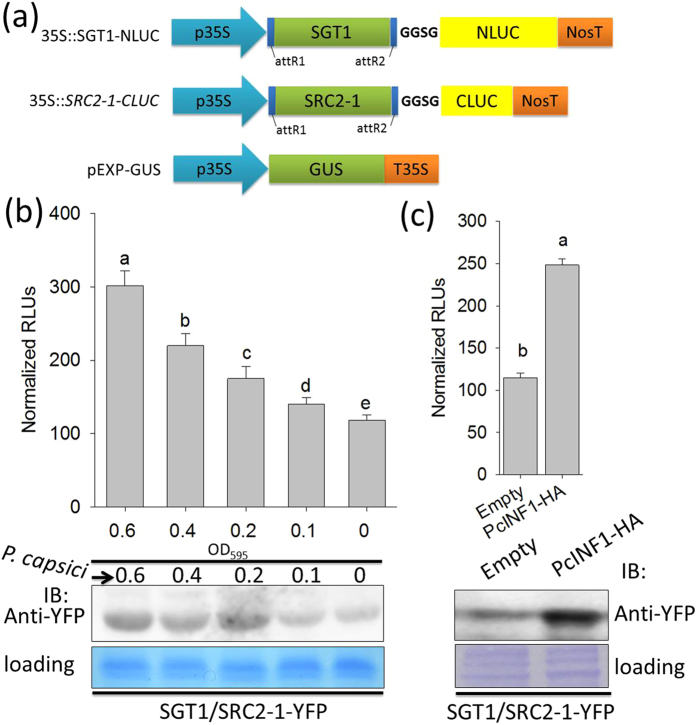
*Phytophthora capsici* and its elicitin PcINF1 triggered the formation of SGT1/SRC2-1 complex in pepper plants. (**a**) Linear structures of the gateway destination vectors of 35::*SGT1-NLUC* and 35S::*SRC2-1-CLUC* for studying their protein interaction using the floated luciferase activity measurement. The pEXP-GUS vector was used for GUS activity measurement to normalize the total protein. (**b**) Analysis of the protein level of SGT1/SRC2-1 complex in pepper leaves infected by *P. capsici* using western-blot (upper) and luminescence activity measurement assay (bottom). GV3101 cells containing the BiFC vector, SGT1/SRC2-1-YFP, were infiltrated into the leaves of pepper plants (OD_595_ = 0.3) and the infiltrated plants were growing in the greenhouse for 48 h, followed by infected with different densities of *P. capsici* zoospores. The total protein of the pathogen-inoculated pepper leaves were extracted for protein levels detection. For the luminescence activity assay, GV3101 cells carrying the split-LUC vectors (35S::*SGT1-NLUC* and 35S::*SRC2-1-CLUC*) were vacuum-infiltrated into the leaves of pepper plants and maintained in the greenhouse for 24 h, followed by *P. capsici* inoculation. The inoculated pepper leaves were harvested for LUC activity measurement at different time points. (**c**) The protein level detection of SGT1/SRC2-1 complex in pepper leaves transiently overexpressing PcINF1-HA and empty vector. The full length ORF of SGT1 and SRC2-1 were first fused to the N-terminal or C-terminal of YPF, respectively, to generate SGT1-YFP^C^ and SRC2-1-YFP^N^. Fragments of SGT1-YFP^C^ and SRC2-1-YFP^N^ in satellite vectors were together recombined into the BiFC destination vector PE3519 to generate SGT1/SRC2-1-YFP. Healthy pepper leaves were first *Agro*-infiltrated with the BiFC vector, SGT1/SRC2-1-YFP, and maintained in the greenhouse for 48 h, followed by *Agro*-infiltration with agrobacterial cells containing the PcINF1-HA and empty vector (OD_595_ = 0.3). The anti-GFP antibodies were used to detect the SGT1/SRC2-1 complex. (**b,c**) The total protein of the treated pepper leaves was extracted and the anti-GFP antibodies were used to detect the SGT1/SRC2-1 complex. Coomassie brilliant blue staining of the membrane was to show the equal loading. Data represent means ± SD for eight independent experiments. Different letters above the bars indicate significantly different means as determined by Fisher’s protected LSD test: lowercase letters, P < 0.05; uppercase letters, P < 0.01.

The functional relationship and the direct interaction between SRC2-1 and SGT1 suggest the involvement of a SRC2-1/SGT1 complex in the defense response triggered by PcINF1. To test its possible mode of action, the interaction between SRC2-1 and SGT1 was detected by assays of luciferase complementation imaging (FLuCI)^7^, immunoblot and fluorescent signals detection under microscope in pepper leaves inoculated with *P. capsici*. For the luciferase complementation imaging assay, the GV3101 cells carry the split-LUC vector (35S::*SGT1-NLUC* and 35S::*SRC2-1-CLUC*) were co-infiltrated into the pepper leaves, followed by inoculated with *P. capsici*. The result showed that the LUC activities was increased in response to *P. capsici* inoculation (Fig. 7a/b), which was also confirmed by immunoblot result as well as the result in fluorescent signals detection under microscope (Fig. 7 and Supplementary Fig. S4). Additionally, the interaction intensity was also found to be enhanced with the increase of dosage of inoculated *P. capsici* (Fig. 7b and Supplementary Fig. S4A). Consistently, the interaction intensity between SRC2-1 and SGT1 was also found to be enhanced by transient overexpression of *PcINF1* in pepper plants (Fig. 7c and Supplementary Fig. S4B).

### The effect of *SGT1*-silencing on PcINF1/SRC2-1 interaction

**Figure 8:**
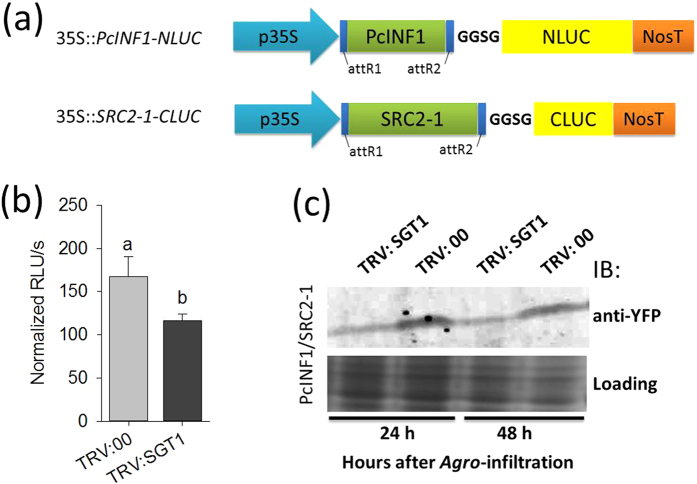
The effect of *SGT1*-silencing on PcINF1/SRC2-1 interaction. (**a**) Linear structure of the gateway destination vectors 35::*SGT1-NLUC*, and 35S::*SRC2-1-CLUC* for studying SGT1/SRC2-1 interaction using the floated luciferase activity measurement. Vector pEXP-GUS vector was used for GUS activity measurement to normalize the total protein. (**b**) The effect of *SGT1-*silencing by VIGS on the PcINF1/SRC2-1 interaction by luciferase complementation analysis. Luminescence values were measured in the *SGT1*-silenced and unsilenced pepper leaves transiently expressing 35S::*PcINF1-NLUC* and 35S::*SRC2-1-CLUC* 48 h post infiltration. Data represent means ± SD for eight independent experiments. Different letters above the bars indicate significantly different means as determined by Fisher’s protected LSD test: lowercase letters, P < 0.05; uppercase letters, P < 0.01.(**c**) The effect of *SGT1-*silencing by VIGS on the PcINF1/SRC2-1 interaction by western-blot analysis. GV3101 cells harboring the PcINF1/SRC2-1 construct were infiltrated into the leaves of *SGT1*-silenced and unsilenced pepper plants. The protein level of PcINF1/SRC2-1 was detected using the western-blot. Anti-GFP antibodies were used to detect the PcINF/SRC2-1-YFP complex. Coomassie brilliant blue staining of the membrane was to show the equal loading.

Since the interaction of SRC2-1 with PcINF1 is required in the defense response triggered by PcINF1^14^. To test if PcINF1/SRC2-1 interaction is affected by SGT1, the effect of *SGT1* silencing on PcINF1/SRC2-1 formation was assayed by luciferase complementation analysis and co-IP. For the luciferase complementation analysis, the GV3101 cells carrying the split-LUC vectors (35S::*PcINF1-NLUC* and 35S::*SRC2-1-CLUC*) were co-infiltrated into the leaves of SGT1-silenced pepper plants and kept in the greenhouse, 24 h after infiltration, the pepper leaves were harvested for LUC activity assay. The result showed that the interaction intensity of SGT1 and SRC2-1 was significantly decreased in the *SGT1*-silenced pepper leaves compared to the control plants (Fig. 8a/b). For the immunoblot assay, GV3101 cells containing PcINF1/SRC2-1-YFP vector (BiFC vector) were infiltrated into leaves of *SGT1*-silenced pepper plants generated by VIGS, which were harvested at 48 HAI for protein extraction. The extracted proteins were separated by SDS-PAGE, after which the PcINF1/SRC2-1 complex was detected by immunoblotting with anti-GFP antibody (sigma). This result showed that the interaction between PcINF1 and SRC2-1 was decreased by the silencing of *SGT1* (Fig. 8c).

### Translocation of SGT1/SRC2-1 complex from membrane to nucleus in *P. capsici* inoculated *N. benthamiana* leaves

**Figure 9:**
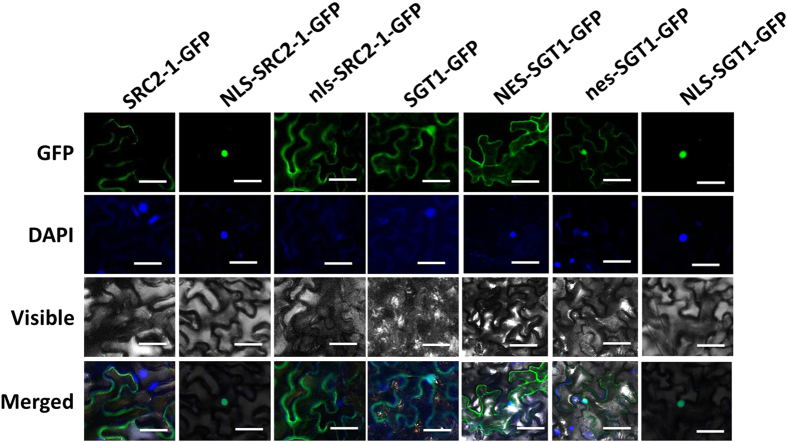
The artificial modulation of subcellular localization of SGT1 and SRC2-1 in cells of *N. benthamiana* leaves. The NLS and NES were employed to artificially target SRC2-1 and SGT1 exclusively to the nuclei and cytoplasm, respectively. GV3101 cells containing the SRC2-1-GFP, NLS(nls)-SRC2-1-GFP, SGT1-GFP, NLS-SGT1-GFP, and NES (nes)-SGT1-GFP vectors were infiltrated into the leaves of *N. benthamiana* plants. 36 h post infiltration, the leaves were harvested and immersed in the DAPI solution and incubated in 37 °C for 1 h and covered with underslip and the fluorescent signals were detected using the Leica confocal laser scanning microscope. Scale bars = 50 μm.

Agrobacterial cells containing constructs of 35S::*SGT1-GFP* or 35S::*SRC2-1-GFP* were infiltrated into *N. benthamiana* leaves, and the subcellular localization of SGT1-GFP and SRC2-1-GFP fused proteins in *N. benthamiana* leaves was detected using confocal microscopy. Consistent with the previous study^14^,^15^, the results show that SRC2-1 was invariably targeted to the membrane of *N. benthamiana* cells, whereas the fluorescent signal from SGT1-GFP in *N. benthamiana* leaves was found throughout whole cells, including in the plasma membrane, cytoplasm, and nucleus (Fig. 9). The observed subcellular localization of SGT1 and SRC2-1 suggests the potentiality for interaction. Bimolecular fluorescence complementation (BiFC) analysis of SGT1 and SRC2-1 interaction in *N. benthamiana* leaves challenged with *P. capsici* inoculation illustrated dynamic localization of the SGT1/SRC2-1 complex. At 24 hours post inoculation (hpi), YFP signal observation showed that among the 30 cells that displayed YFP signals, 28 cells exhibited YFP localization in plasma membrane or cytoplasm except for the nuclei, while at 48 and 72 hpi, 27 and 28 cells exhibited their YFP signals in the whole cell including the nuclei among the 30 checked cells with YFP signals, respectively (Supplementary Figs 5–6). In addition, we also detected the protein levels of SRC2-1 in the cytoplasm and the nuclei in *N. benthamiana* leaves with or without co-expression of SGT1, the result showed that SRC2-1 protein was detected only in the cytoplasm when it expressed alone (Supplementary Fig. S7), but was detected in both of the cytoplasm and the nuclei when SGT1 was co-expressed (Supplementary Fig. S7).

### Exclusive nucleus or cytoplasm localization of SGT1/SRC2-1 impaired cell death in pepper plants

To test the possible role of SGT1/SRC2-1 complex translocation from membrane to nucleus in plant immunity, the localization of SGT1/SRC2-1 complex was modified using nuclear localization signal (NLS)^45^ and nuclear export signal (NES)^22^. Constructs of *SRC2-1* fused to *NLS* and *SGT1* fused to *NES*, termed 35S::*NLS-SRC2-1-GFP* and 35S::*NES-SGT1-GFP*, were generated, and a nonfunctional NES mutant (nes) and a NLS mutant (*nls*) were also constructed and fused to *SGT1* and *SRC2-1*, respectively, to generate 35S::*nes-SGT1-GFP* and 35S::*nls-SRC2-1*-GFP constructs. All of these constructs were transformed into GV3101 cells, and cells containing 35S::*NES-SGT1-GFP*, 35S::*NLS-SRC2-1-GFP*, 35S::*nes-SGT1-GFP*, or 35S::*nls-SRC2-1-GFP* were infiltrated into leaves of *N. benthamiana*. GFP signals were then detected with confocal microscopy. The results demonstrated that one NLS tag was sufficient to target SRC2-1 to the nuclei of *N. benthamiana* leaf cells, and the transient expression of 35S::*NES-SGT1-GFP* construct exhibited an absence of nuclear GFP fluorescence signals in the *N. benthamiana* leaf cells, instead, a visible cytoplasmic GFP fluorescence was observed (Fig. 9).

**Figure 10:**
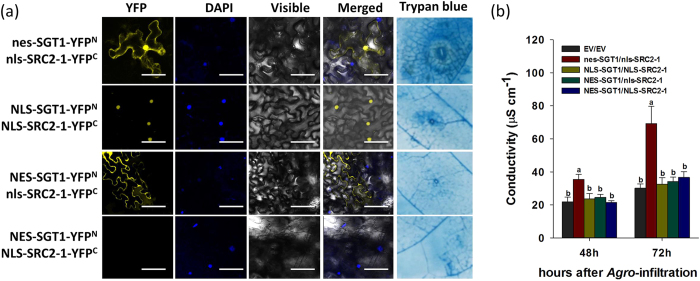
The exclusive nuclear or cytoplasm localization of SGT1/SRC2-1 failed to trigger cell death response in pepper leaves. (**a**) BiFC fluorescence and the cell death detection of NLS-(nls-) or NES-(nes-) fused SGT1/SRC2-1 combinations in *N. benthamiana* leaves 48 h post infiltration. GV3101 harboring the BiFC serial vectors were infiltrated into the leaves of *N. benthamiana* using a needleless syringe and maintained in the climate chamber, which were harvested and immersed in the DAPI solution and incubated in 37 °C for 1 h before fluorescence detection using the Leica laser scanning microscope. Meanwhile, the infiltrated pepper leaves were also harvested for trypan blue staining. Scale bars = 50 μm. (**b**) The electrolyte leakage measurement of pepper leaves infiltrated with GV3101 cells containing NLS-(nls-) or NES-(nes-) fused SGT1/SRC2-1 combinations. The infiltrated pepper leaves were harvested for electrolyte ion measurement. Scale bars = 100 μm. Data represents the mean ± SD from three independent biological repetitions. Different letters indicate statistically significant differences as analyzed with an LSD test (*P* < 0.05).

To test the effect of subcellular localization of the SGT1/SRC2-1 complex on pepper immunity, a set of BiFC vectors that include an NLS tag (*NLS-SRC2-1-YFP*^C^ and *nls-SRC2-1-YFP*^C^) and NES tag (*NES-SGT1-YFP*^N^ *and nes-SGT1-YFP*^N^) were constructed. BiFC analysis using different combinations of vectors was performed, and the corresponding induction of immunity associated with these combinations was also assayed. The results show that YFP signals were detected both in the nuclei and cytoplasm of cells of *N. benthamiana* leaf transiently overexpressing nes-SGT1-YFP^N^/nls-SRC2-1-YFP^C^. However, the YFP signals were detected in the nuclei of *N. benthamiana* leaf cells when *NLS-SGT1-YFP*^N^/*NLS-SRC2-1-YFP*^C^ was simultaneously co-expressed, while the YFP signals were detected in the cytoplasm of *N. benthamiana* leaf cells when NES-SGT1-YFP^N^/nls-SRC2-1-YFP^C^ was simultaneously co-expressed. The YFP signal was not detected after co-expression of *NES-SGT1-YFP*^N^/*NLS-SRC2-1-YFP*^C^ (Fig. 10a). Among the vector combinations that produced YFP signals, induced immunity could only be detected (by trypan blue staining and the electrolyte leakage measurement) in leaf cells co-expressing *nes-SGT1-YFP*^N^/*nls-SRC2-1-YFP*^C^ (Fig. 10a/b).

## Discussion

Despite the important roles of SGT1 and SRC2-1 in plant immunity suggested by previous studies^14^,^40^, a functional relationship between the two proteins and their potential roles in plant immunity have not yet been established. This study provides evidence of a novel interaction between SGT1 and SRC2-1, and a role for this interaction in pepper immunity induced by PcINF1/SRC2-1.

Using real-time PCR analysis, the response of the transcriptional expression of *SGT1* to *P. capsici* inoculation as well as transient overexpression of *PcINF1*, an elicitin from *P. capsici*, was investigated. The qPCR result showed that the mRNA level of *SGT1* can be induced in response to the *P. capsici* inoculation and *PcINF1*/*SRC2-1* co-expression, implying its potential involvement in pepper immunity against *P. capsici*. This potentiality was further confirmed by loss-of-function assay that employed VIGS. The silencing of *SGT1* significantly blocked the cell death and upregulation of immunity associated marker genes in pepper plants challenged with *P. capsici* or in *PcINF1*/*SRC2-1* transiently overexpressed pepper plants, suggesting the involvement of SGT1 in pepper immunity against *P. capsici* and in the defense response of pepper triggered by transient overexpression of *PcINF1*/*SRC2-1*. As PcINF1 is a member of the conserved elicitin family in *P. capsici*, the defense response triggered by PcINF1, which is mediated by SGT1, may contribute to the pepper immunity against PTI.

SGT1 was previously found to act as a positive regulator in both ETI^18^,^19^,^46^,^47^ and PTI pathways^9^. For example, the overexpression of *OsSGT1* in rice significantly increased basal resistance to the virulent bacterial blight *Xanthomonas oryzae* pv. *oryzae* PXO99^9^. In addition to its well-established positive roles, SGT1 has also been found to act as a negative regulator of immune receptor accumulation and is thought to assist in maintaining appropriate levels of immune receptor proteins in order to avoid autoimmunity^48^,^49^. A recent study found that SGT1, as well as PAR and HSP90, negatively regulates bacterial wilt disease caused by *Ralstonia solanacearum* in *N. benthamiana*^10^. Similarly, *SGT1* was found to be induced by the potato virus X TGBp3, and enhanced virus accumulation in *N. benthamiana*^50^. These discrepancies might be due to genetic differences both in pathogens and host plants^51^,^52^. For example, Arabidopsis SGT1b can antagonize RAR1 to negatively regulate R protein accumulation before infection, and can also function in a RAR1-independent manner to regulate PCD during infection^52^. Consequently, SGT1 may function differently in plant-pathogen combinations with different effectors and R proteins.

### SGT1 is required in PcINF1/SRC2-1 induced pepper immunity by interacting to SRC2-1

SRC2-1, a C2 domain containing protein that was found to act as an interacting partner of PcINF1 in its pepper immunity induction^14^, was found to synergistically respond to *P. capsici* inoculation as well as to transient overexpression of PcINF1 with SRC2-1. Also in the present study, the pathogen-responsive W-box and HSE were consistently found to present in the promoters of both SGT1 and SRC2-1 (Supplementary Figs S8/S9 and supplementary Table S3). Given that SGT1 acts as a versatile chaperone, and SRC2-1 contains a C2 domain conferring protein interaction, we speculated that SGT1 and SRC2-1 could potentially be functionally associated in a protein interaction manner. To test this speculation, BiFC experiments were performed and the result potentially demonstrated SGT1 interacts with SRC2-1 which was further confirmed through co-IP experiment, but the potential involvement of other components in the SGT1/SRC2-1 complex cannot be excluded. Additionally, the interaction between PcINF1 and SRC2-1 was found to be decreased by the silencing of *SGT1*; on the other hand, the transient overexpression of PcINF1 and *P. capsici* inoculation significantly enhanced the interaction between SGT1 and SRC2-1. All of these results indicated that SGT1 and SRC2-1 are functionally related in the response of pepper to PcINF1 transient overexpression or *P. capsici* inoculation.

### Nucleocytoplasmic distribution of SGT1/SRC2-1 complex is required for its immunity activation

The function of any given protein may be closely associated with its subcellular localization. Similar to the observations made in a previous study^15^, transiently overexpressed GFP-fused SRC2-1 was exclusively localized to the plasma membrane in *N. benthamiana* leaf epidermis cells, whereas SGT1 was localized throughout the cells including in the plasma membrane, cytoplasm, and nucleus in the present study. Similar observations of SGT1 were also previously reported in other plant species^53^. Given that no NLS is found in SGT1, its nuclear targeting is possibly due to the interaction with other nuclear-targeting proteins. Indeed, SGT1 acts as a general adaptor protein in different plant species, interacting with various classes of proteins including R proteins [Prf^54^ and NLR^55^], other chaperones [OsCYP2^56^, Hsp90^32^,^57^, RAR1^9^, receptor-like cytoplasmic kinase^38^,^40^, and type III effector AvrBsT^6^]. Nucleocytoplasmic distributions have been recently found for TIR-NB-LRRs and CC-NB-LRRs receptors, such as N protein and Rx, which are modulated by SGT1 and are required for full disease resistance^22^,^23^,^27^. Our data showed that this nucleocytoplasmic distribution was also displayed for the SGT1/SRC2-1 interaction complex, as illustrated through its translocation from the membrane and cytoplasm into the nuclei in BiFC assays. By using the NLS and NES-tagged proteins to artificially modify the subcellular localization of SRC2-1 and SGT1, we found the exclusive nuclear or cytoplasmic targeting of SGT1/SRC2-1 complex significantly impaired pepper immunity. This result suggests that the translocation of the SGT1/SRC2-1 complex from the cytoplasm to the nucleus is required for the pepper’s immunity against *P. capsici*, and this nucleocytoplasmic portioning is potentially mediated by other proteins, such as HSC70 and HSP90, which play roles in nucleocytoplasmic shuttling in several eukaryotic systems^58^,^59^,^60^,^61^,^62^, since SGT1 can interact with these proteins by previous studies^44^,^59^,^63^,^64^. Similarly, the PcINF1/SRC2-1 complex was also found to have a nucleocytoplasmic distribution by our previous study^14^. It is potential that this nucleocytoplasmic portioning may also be mediated by HSC70 or HSP90. Further experiments are needed to elucidate the potential involvement of HSC70 or HSP90 in SGT1/SRC2-1 nucleocytoplasmic balancing.

Collectively, the data in this study suggest that SGT1 is required in PcINF1/SRC2-1 induced pepper defense response by interacting with SRC2-1, and that the nucleocytoplasmic partitioning of the resulting protein complex is required for plant immunity.

## Methods

### Plant materials and pathogen inoculation

Pepper cultivar (*Capsicum annuum* L. cv *yanshan01*) was collected from Yanshan County, Yunnan Province, China. The seeds of *N. benthamiana* were stored in our lab. The *Phytophthora capsici* strain used in this study was isolated from *Phytophthora* blight pepper plants in Huian County, Fujian Province, China. The pepper and *N. benthamiana* plants were grown as previously described^66^. For inoculation, *P. capsici* was cultured in V8 medium (200 g l^−1^ tomato juice, 3 g l^−1^ CaCO_3_) overnight at 28 °C. *P. capsici* was collected with low speed centrifugation, suspended in sterile ddH_2_O, and then adjusted to a density of 100 zoospores ml^−1^. The zoospore suspension of *P. capsici* was then infiltrated into the lower epidermal of the pepper leaves using a syringe without a needle. The *P. capsici*-infected pepper plants were then transferred into a climate chamber at 28 °C with 100 mmol photons m^−2^ s^−1^, a relative humidity of 70%, and a 16-h light/8-h dark cycle.

### Quantitative real-time PCR assay

The pepper leaves collected from treatments were frozen in liquid nitrogen immediately and stored in the −80 °C freezer. The frozen leaf tissues were disrupted in 1.5 ml RNAse-free microcentrifuge tubes using 3 stainless steel beads and the Tissue Lyser II (Qiagen, Dusseldorf, Germany). Trizol (Invitrogen, Carlsbad, USA) was used to extract the total RNA from the disrupted tissues. Both the concentration and the quality of the total RNA were detected using the NanoDrop 2000 (ThermoScientific, Massachusetts, USA). 500 ng of RNA from each sample was used for the first cDNA strand generation using One Step PrimeScript^TM^ cDNA Synthesis Kit (TaKaRa, Shigo, Japan), which include a genomic DNA digestion step according to the manufacturer’s instructions (TaKaRa). The synthesized cDNA products were diluted ten-fold in sterilized RNAse-free ddH_2_O for further qPCR analysis. qPCR was performed as a 20-μL reaction (10 μL 2× SYBR Premix Ex-Taq, 0.2 μM forward and reverse primers, and 1 μL cDNA) in a 200-μL PCR tube using SYBR Premix Ex Taq^TM^ (TaKaRa) and a CFX^TM^ Real-Time System with the following program: 95 °C, 30 s; 95 °C, 5 s; 60 °C, 34 s; 95 °C, 15 s, 40 cycles. SYBR green fluorescence was used to detect the amplification of the target genes at each cycle. The Cq (threshold cycle), defined as the real-time PCR cycle at which a statistically significant increase of reporter fluorescence was first detected, was used as a measure for the starting copy numbers of the target gene. Three replicates of each experiment were performed. The data were analyzed by the Livak method^70^ and expressed as a normalized relative expression level (2^−ΔΔCT^) of the respective genes. *CaActin* (GQ339766) and 18S ribosomal RNA (EF564281), which have been confirmed by the published paper^66^,^67^, were used to normalize the transcript levels in pepper. The specificities of the qPCR primers were confirmed by the melting curve and the qPCR validation was investigated using the Bio-rad CFX manager 2.0 (Supplementary Table S1). All gene-specific primers are listed in Supplementary Table S1.

### Bimolecular fluorescence complementation (BiFC) analysis

To construct vectors for BiFC analysis, the open reading frames (ORFs) of *SGT1* and *SRC2-1* (flanked by the attB sequence) were amplified with gene-specific primer pairs listed in Supplementary Table S2. Products were introduced into pDONR207 to yield pDONR vectors (named *SGT1*-207 and *SRC2-1*-207), and then transferred into the satellite vectors pE3130 and pE3136 to generate SGT1-YFP^N^ and SRC2-1-YFP^C^. The reciprocal constructs, SGT1-YFP^C^ and SRC2-1-YFP^N^, were constructed similarly. Fragments of SGT1-YFP^N^ and SRC2-1-YFP^C^ or SGT1-YFP^C^ and SRC2-1-YFP^N^ in satellite vectors were together recombined into the destination vector PE3519, which was then transformed into the *Agrobacterium* strain GV3101. Agrobacterial cells harboring the BiFC constructs were *Agro*-infiltrated into the leaves of *N. benthamiana* plants. Two days after *Agro*-infiltration, fluorescent signals from *N. benthamiana* leaves were analyzed using a confocal microscope DM6000 CS (Leica, Solms, Germany). For DAPI staining, *N. benthamiana* leaves were immersed in DAPI solution (0.1% DAPI and 5% DMSO) and incubated at 37 °C for 1 h before observation.

### Generation of Gateway-compatible split-LUC vectors and LUC activity measurement

LUC destination vectors used in this work are purchased using the forms provided at http://www.uni-muenster.de/Biologie.IBBP/agkudla/Plasmids.html. To construct split-LUC vectors, the attB-flanked open reading frames of SGT1 and SRC2-1 without stop codon were amplified with gene-specific primers and the PCR products were introduced into the satellite vector pDONR207. After sequencing confirmation, the SGT1 (PcINF1) and SRC2-1 entry vectors were transformed into the destination vectors (pDEST-^GW^NLUC and pDEST-^GW^CLUC) to generate 35S::*SGT1-NLUC* (35S::*PcINF1-NLUC*) and 35S::*SRC2-1-CLUC*, respectively. The yielded SGT1- and SRC2- LUC vectors were transformed into the *Agrobacterium* stain GV3101. GV3101 cells harboring the various split-LUC constructs were used for infiltration in pepper leaves. GV3101 cells carrying the indicated constructs were centrifuged and resuspended in the infiltration buffer (10 mM MgCl_2_, 10 mM MES, 150 μM acetosyringone) at an OD595 of 0.4 for LUC activity measurement. Subsequently, the indicated split-LUC constructs and pEXP-GUS were mixed at 1:1 ratio and incubated with gentle shaking at room temperature for 4 hours before infiltration into young but fully expanded leaves of 6–7 week-old pepper plants. After infiltration, the pepper plants were maintained in the greenhouse until LUC activity measurement. Leaf pairs of each pepper plants were infiltrated with the sample in one leaf and the corresponding negative control in the other leaf. Luminescence was quantified in pepper leaves using a floated leaf disc assay^15^ at appropriate times after infiltration. LUC assay solution (0.1 mM luciferin dissolved in 0.5% v/v DMSO, 10 mM MgCl_2_ and 10 mM MES/KOH, pH 5.6) was prepared and infiltrated into the agro-infiltrated-pepper leaf before being cut out as leaf discs of 12 mm diameter. The leaf discs were placed in the 96-well microplate with the same LUC assay solution. The light production was measured by a microplate reader (Biotek; http://www.biotek.com). Before LUC activity measurement, the pepper plant were kept in the dark for 10 min to minimize the background fluorescence, and infiltration of LUC assay solution was performed in the low-light condition.

### GUS activity measurement

To normalize the LUC values for different expression levels in pepper leaves, GUS activity measurement was performed. Before *Agro*-infiltration, all the GV3101 cells carrying the indicated constructs were co-infiltrated with a GUS expression vector (pEXP-GUS). To quantitatively measure GUS activity, protein extraction buffer [10% glycerol, 25 mM Tris-HCl, pH 7.5, 150 mM NaCl, 1 mM EDTA, 2% Triton X-100, 10 mM DTT, 1× complete protease inhibitor cocktail (Sigma-Aldrich; http://www.sigmaaldrich.com), and 2% (w/v) polyvinylpolypyrrolidone]^16^ was used to extract the total protein of infiltrated pepper leave. To measure GUS activity, the rate of *p*-nitrophenol (γ = 415 nm) release was quantified using a microplate reader (Biotek; http://www.biotek.com).

### Yeast two hybrid assay

The GAL4-based Y2H library screening system was performed according to the manufacturer’s instructions (Invitrogen). The PCR-amplified, attb-containing ORFs of *SGT1* and *SRC2-1* were introduced into the gateway-compatible vectors pDONR201 and pDONR207, respectively, to generate entry vectors. The entry vectors were then confirmed by sequencing and transformed into the bait vector pDEST32 and the prey vector pDEST22 to generate BD-SGT1 and AD-SRC2-1, respectively. We transformed these constructs into yeast strain MaV203 with positive-control and negative-control vectors and were allowed to grow in the selection media (SD-Leu-Trp-His/+ appropriate concentrations of 3-amino-1, 2, 4-triaole).

### Co-immunoprecipitation (Co-IP) assay

To construct vectors for co-IP analysis, *SGT1* or *SRC2-1* in the pDONR vector was directly introduced into the destination vectors 35S::Flag (pEarleyGate 202) or 35S::6HA (pEarleyGate 201) to generate 35S::*SGT1-Flag* and 35S::*SRC2-1-HA* by LR reaction (Invitrogen). Plasmids were transformed into the *Agrobacterium* strain GV3101, and cells harboring 35S::*SGT1-Flag* and 35S::*SRC2-1-HA* were simultaneously infiltrated into leaves of *N. benthamiana* plants. Leaves were harvested at 48 hours after infiltration (HAI) and total proteins were extracted using protein extraction buffer [10% glycerol, 25 mM Tris-HCl, pH 7.5, 150 mM NaCl, 1 mM EDTA, 2% Triton X-100, 10 mM DTT, 1× complete protease inhibitor cocktail (Sigma-Aldrich, St. Louis, MO, USA), and 2% (w/v) polyvinylpolypyrrolidone]^69^. Extracted proteins were incubated with monoclonal anti-HA magnetic beads (Sigma-Aldrich) at 4 °C overnight. Beads were then collected with a magnet and washed three times with protein extraction buffer. Eluted proteins were immunoblotted using anti-HA-peroxidase antibody or anti-Flag-peroxidase antibody (Sigma-Aldrich).

### *Agrobacterium*-mediated transient overexpression and subcellular localization assay

To construct vectors for transient overexpression of *SGT1* and *SRC2-1*, *SGT1* and *SRC2-1* from plasmids constructed for BiFC experiments (previously described) were transferred into gateway-compatible destination vectors pK7WG2 and pMDC45 (Invitrogen) using LR reaction to yielded pK7WG2-*SGT1,* pK7WG2-*SRC2-1,* pMDC45-*SGT1*, and pMDC45-*SRC2-1*. All of these vectors were transformed into the *Agrobacterium* strain GV3101, and agrobacterial cells containing the appropriate vectors were streaked on solid YEP medium (10 g Bacto peptone, 10 g yeast extract, 5 g NaCl, and 15 g agar in 1 l of water) and grown at 28 °C. Individual clones were selected two days later and further cultivated in YEP liquid medium containing appropriate antibiotics. Cells in liquid medium were collected and then suspended in MMA infiltration buffer (10 mM MgCl_2_, 10 mM MES, 200 μm acetosyringone). The MMA-suspended cells were infiltrated into the full leaves of pepper or *N. benthamiana* plants using a syringe without a needle.

For subcellular localization assays, the plasmids described above were used. Green fluorescence in *N. benthamiana* leaves infiltrated with GV3101 cells containing pMDC45-*SGT1* and pMDC45-*SRC2-1* was observed using a fluorescence microscope Eclipse Ni-U (Nikon, Tokyo, Japan) at 48 HAI. For visualization of nuclei, leaf samples were stained in DAPI solution and incubated at 37 °C for 1 h before observation.

### Nuclear targeting of SRC2-1 and nuclear export of SGT1 using a nuclear localization sequence (NLS) peptide and a nuclear export sequences (NES)

To artificially target SRC2-1 to the nuclei, DNA for a general NLS (5′-GGCCCTAAAAAGAAGCGTAAGGTT-3′)^45^ was fused to DNA corresponding to the N-terminal of the ORF of *SRC2-1*. A negative control sequence (nls) was produced by substituting an Asn residue for a Lys (5′- GGCCCTAAAAACAAGCGTAAGGTT-3′)^45^. To drive nuclear export of SGT1, an NES (CTTGCATTAAAGCTCGCTGGTCTTGATATT) fragment was fused to the N-terminal of the ORF of SGT1. The NLS- and NES-fused ORF of *SGT1* and *SRC2-1* was first cloned into the pDONR207, sequenced and transferred into the corresponding destination vectors, and eventually introduced into the agrobacterial cells for further study.

The nuclear and cytoplasmic proteins were extracted using the Eukaryotic Nuclear Protein Extraction Kit or Eukaryotic Membrane Protein Extraction Kit, respectively (Thermo scientific).

### Virus-induced gene silencing (VIGS) in pepper plants

VIGS technology was used to study the loss-function of *SGT1* and *SRC2-1*. The VIGS assays were performed as described previously^14^. To re-describe the method briefly, specific 3′ UTR fragments of *SGT1* and *SRC2-1* around 200 bps in length were amplified and cloned into the vector pTRV2 using the primers listed in Supplementary Table S2. These vectors were then transformed into GV3101 cells, and agrobacterial cells harboring the TRV-derived vectors (TRV1/TRV2-SGT1, TRV1/TRV2-SRC2-1, TRV1/TRV2- SGT1/SRC2-1, and TRV1/empty) were streaked onto solid YEB medium and grown at 28 °C. Individual clones were picked two days later and cultured in liquid YEP medium containing 75 μg ml^−1^ kanamycin and 75 μg ml^−1^ rifampicin. GV3101 cells were collected by centrifugation and suspended in MMA infiltration buffer (10 mM MgCl_2_, 10 mM MES, 200 μM acetosyringone, pH 5.7), and adjusted to an OD_595_ of 0.6. Suspensions of GV3101 cells containing TRV1/TRV2-SGT1, TRV1/TRV2-SRC2-1, TRV1/TRV2-SGT1/SRC2-1, or TRV1/empty were mixed at the ratio of 1:1 and incubated at room temperature in a shaker at low speed (50 rpm). After gentle shaking for 3 h, the GV3101 cells containing vectors in each combination were co-infiltrated into the cotyledons of 2-week-old pepper seedlings.

### Conductivity leakage measurements

The conductivity of leaves was analyzed by measuring ion leakage following the method of Choi *et al.*^70^ with slight modification. Six leaf discs (4 cm in diameter) were cut with a hole punch, washed with sterilized ddH_2_O for three times, and subsequently incubated in 20-ml volume of double-distilled water and maintained in a shaker with gently shaking (at 60 rpm) for 60 min at room temperature. The ion leakage of the bathing solution was measured with a conductivity meter (Mettler Toledo 326 Mettler, Zurich, Switzerland).

### DAB and trypan blue staining

DAB (3, 3′-diaminobenzidine) and trypan blue staining were performed after *Agro*-infiltration using the method^70^ to visualize H_2_O_2_ accumulation and cell death. For DAB staining, the full *Agro*-infiltrated leaves were immersed in DAB solution (1 mg ml^−1^ DAB, pH 5.7), protected from light and incubated overnight (for almost 8 h). Leaves were then de-stained with 95% ethanol three times. For trypan blue staining, the full *Agro*-infiltrated leaves were boiled in a lactophenol-ethanol trypan blue solution (10 ml lactic acid, 10 ml glycerol, 10 g phenol, 30 ml absolute ethanol, and 10 mg of trypan blue dissolved in 10 ml of distilled water) for 5 min and allowed to rest at room temperature overnight. Leaves were de-stained with 2.5 g ml^−1^ chloral hydrate in distilled water.

## Additional Information

**How to cite this article**: Liu, Z.-q. *et al.* SGT1 is required in PcINF1/SRC2-1 induced pepper defense response by interacting with SRC2-1. *Sci. Rep.* **6**, 21651; doi: 10.1038/srep21651 (2016).

## Supplementary Material

Supplementary Information
