# Supplementary material for: SGT1 is required in PcINF1/SRC2-1 induced pepper defense response by interacting with SRC2-1
Source: Sci Rep. 2016 Feb 22;6:21651. doi: 10.1038/srep21651 (PMC4761932; doi:10.1038/srep21651)
Supplement: Supplementary Information [file srep21651-s1.pdf]

SGT1 is required in PcINF1/SRC2-1 induced pepper defense response by interacting with SRC2-1

Zhi-qin Liu[1,2,†], Yan-yan Liu[1,2,†], Lan-ping Shi[1,2,†], Sheng Yang[1,2], Lei Shen[1,2], Huan-xin Yu[1,2], Rong-zhang Wang[1,2], Jia-yu Wen[1,2], Qian Tang[1,2], Ansar Hussain[1,2], Muhammad Ifnan Khan[1,2], Jiong Hu[1,2], Cai-ling Liu[1,2], Yang-wen Zhang[1,2], Wei Cheng[1,2], and Shui-lin He[1,2, *]

[†]These authors contributed equally to this study

[*]To whom correspondence should be addressed. Shuilin He: shlhe201304@aliyun.com

Supplementary figures

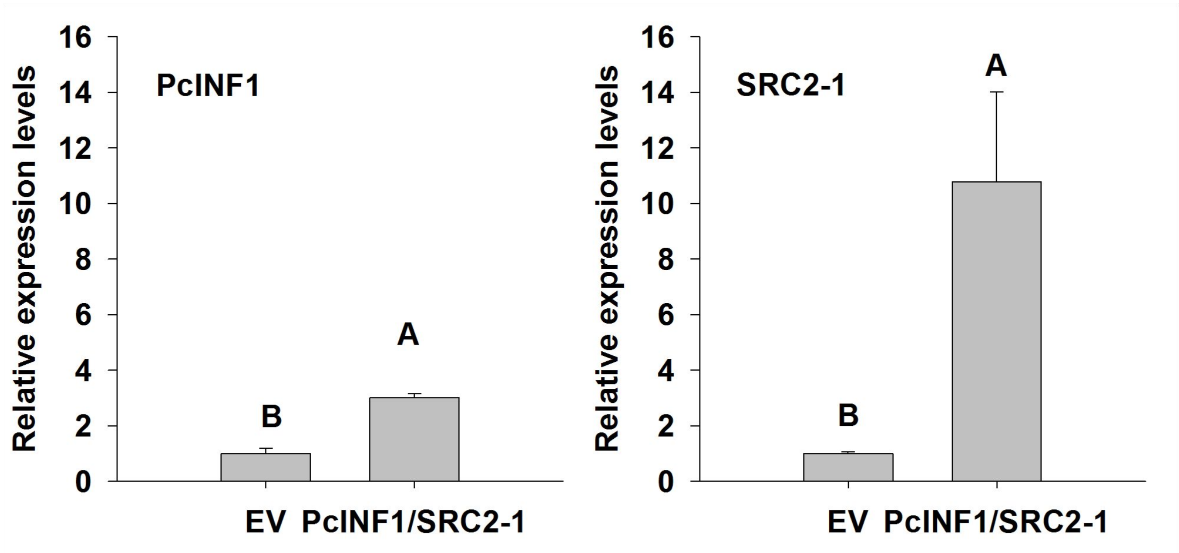

Fig. S1. qPCR analysis of the transcript levels of *PcINF1* and *SRC2-1* in pepper plants 24 h after transiently overexpressing *PcINF1* and *SRC2-1*. Transcript values are normalized to the mRNA level of *CaActin* and 18S ribosomal RNA. Data represent the mean ± standard error from three independent experiments. Different letters indicate statistically significant differences, as analyzed using Fisher's protected least significant difference (LSD) test ($P < 0.05$). Mock, treated with 10 mM $MgCl_2$.

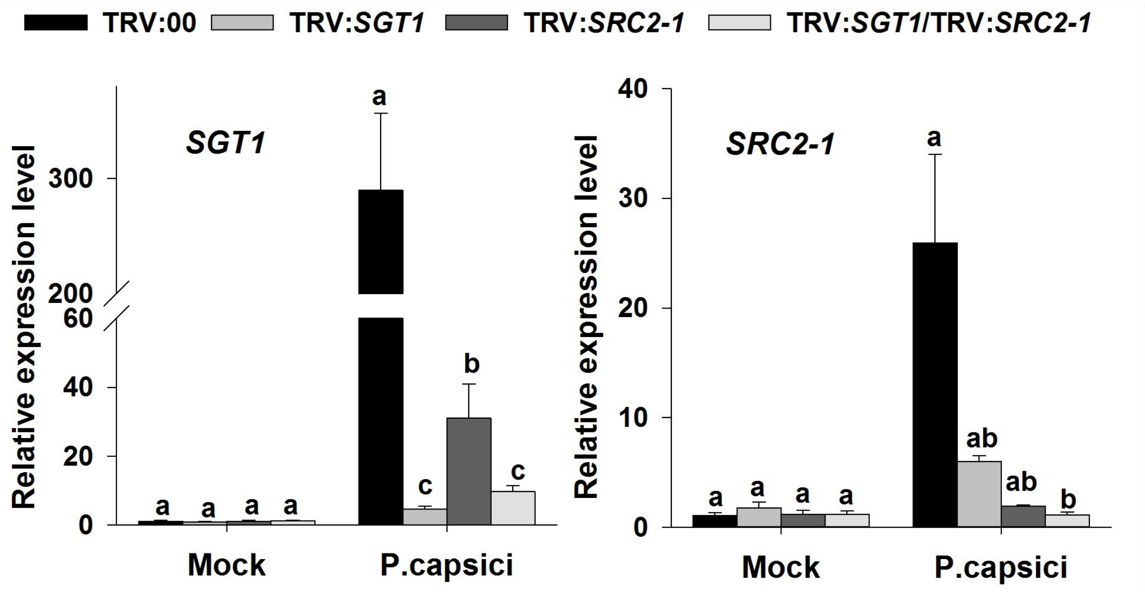

Fig. S2. qPCR analysis of the transcript levels of *SGT1* and *SRC2-1* in TRV:00 and VIGS pepper plants 24 h after inoculation with *P. capsici*. Transcript values are normalized to the mRNA level of *CaActin* and 18S ribosomal RNA. Data represent the mean ± standard error from three independent experiments. Different letters indicate statistically significant differences, as analyzed using Fisher's protected least significant difference (LSD) test ($P < 0.05$). Mock, treated with 10 mM $MgCl_2$.

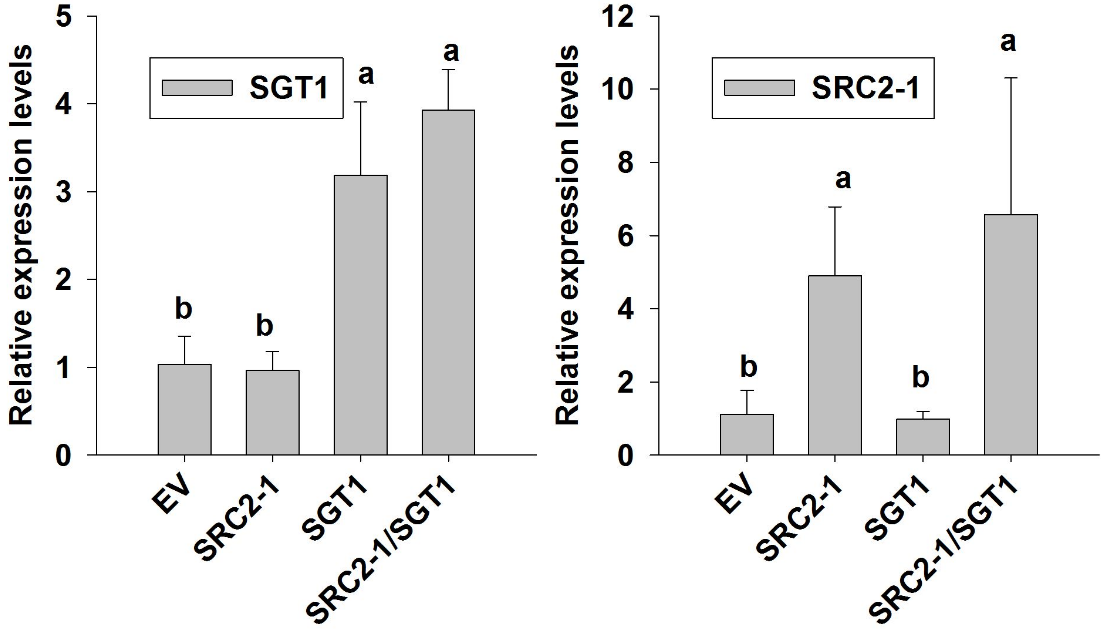

Fig. S3. qPCR analysis of the transcript levels of *SGT1* and *SRC2-1* in pepper plants 24 h after transiently overexpressing *SGT1* and *SRC2-1*. Transcript values are normalized to the mRNA level of *CaActin* and 18S ribosomal RNA. Data represent the mean $\pm$ standard error from three independent experiments. Different letters indicate statistically significant differences, as analyzed using Fisher's protected least significant difference (LSD) test ($P < 0.05$). Mock, treated with 10 mM $MgCl_2$.

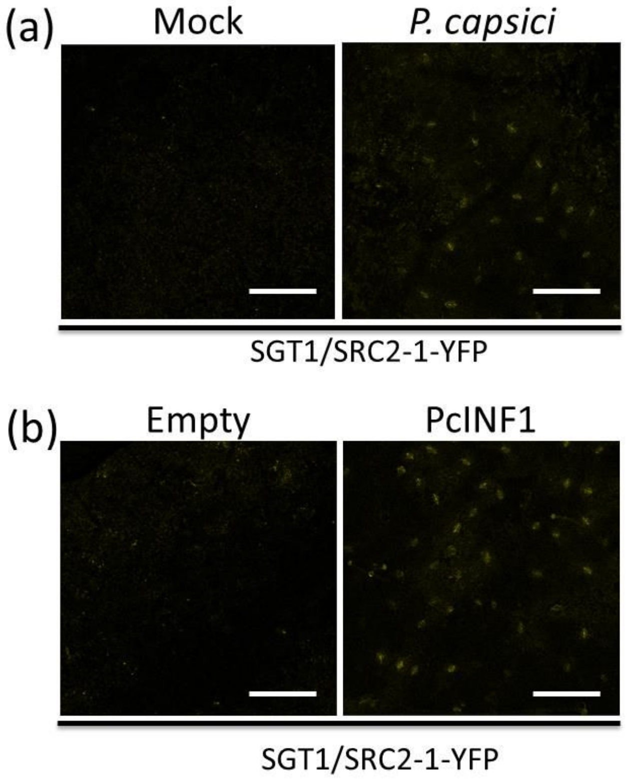

Fig. S4. *Phytophthora capsici* and its elicitin PcINF1 potentiate the formation of SGT1/SRC2-1 complex in *N. benthamiana* leaves detected in the microscope. Agrobacteral cells (OD595=0.4) carrying the SGT1/SRC2-1-YFP were vacuum-infiltrated into the leaves of *N. benthamiana* and maintained in the greenhouse. 24 h after infiltration, the infiltrated leaves were inoculated with *Phytophthora capsici* or its elicitin PcINF1.

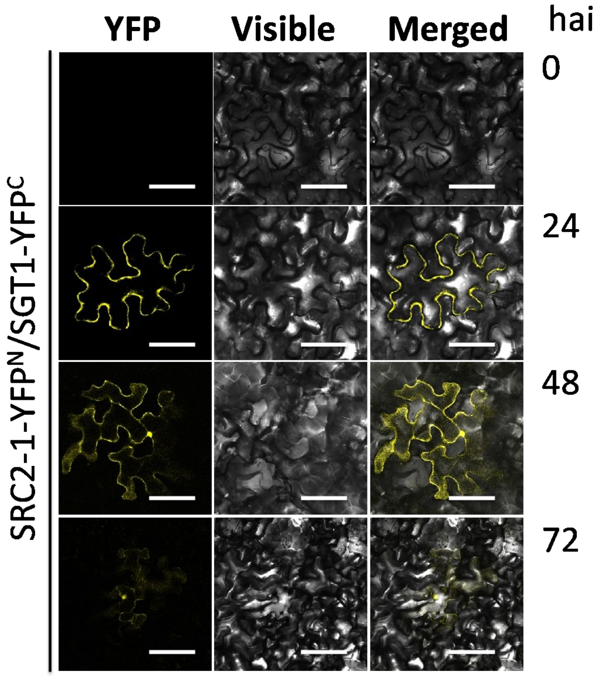

Fig. S5. SGT1 and SRC2-1 interact in the membrane and translocate into the cytoplasm after *P. capsici* inoculation. BiFC was used to visualize the SGT1/SRC2-1 interaction in *N. benthamiana* leaves inoculated with *P. capsici*. Yellow fluorescence, visible light and merged images were taken of epidermal cells infiltrated with a mixture of *Agrobacterium* suspensions harboring the constructs encoding SGT1-YFP^C and SRC2-1-YFP^N fusion proteins. Time course images were obtained with confocal microscopy after *Agro*-infiltration. Scale bars = 100 µm. Hai, hours after inoculation.

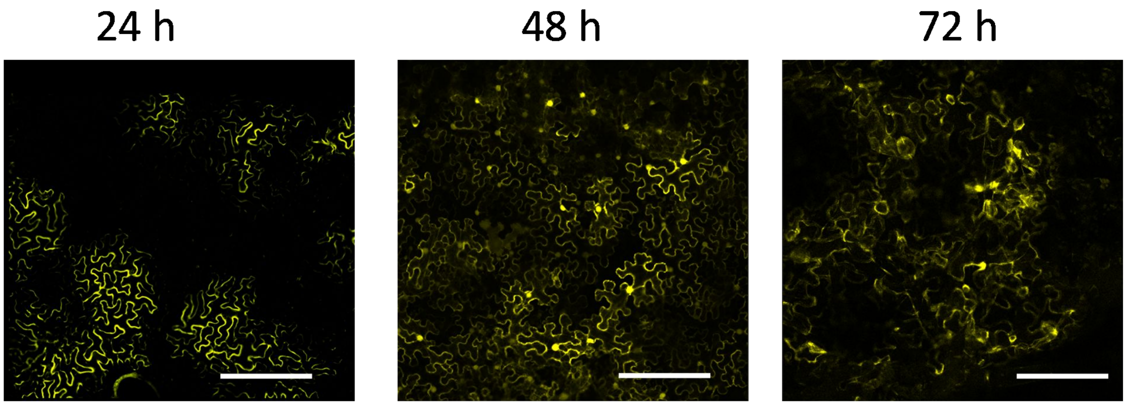

Fig. S6. SGT1 and SRC2-1 interact in the membrane and translocate into the cytoplasm after *P. capsici* inoculation. BiFC was used to visualize the SGT1/SRC2-1 interaction in *N. benthamiana* leaves inoculated with *P. capsici*. Yellow fluorescence, visible light and merged images were taken of epidermal cells infiltrated with a mixture of *Agrobacterium* suspensions harboring the constructs encoding SGT1-YFP^C and SRC2-1-YFP^N fusion proteins. Time course images were obtained with confocal microscopy after *Agro*-infiltration. Scale bars = 200 µm.

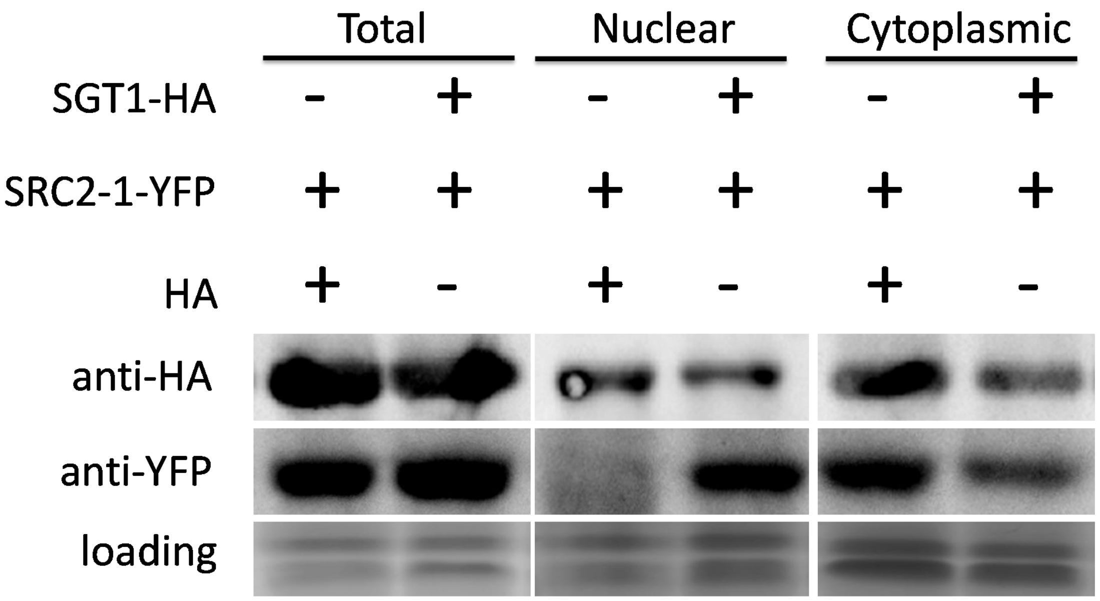

Fig. S7. SGT1 triggers SRC2-1 translocate from membrane to nulcear. Immunoblot analysis of the protein levels of SRC2-1 and SGT1 in pepper leaves transiently expressing SGT1-HA with or without SRC2-1-YFP. Agrobacteral cells carrying the 35::SGT1-HA or 35S::SRC2-1-YFP were infiltrated into the pepper leaves . The cytoplasmic and nuclear protein of the infiltrated leaves were extracted 48 h after Agro-infiltration. Anti-HA and anti-YFP antibodies were used to detect SGT1-HA and SRC2-1-YFP proteins. Coomassie brilliant blue staining of the membrane was to show the equal loading

SGT1 promoter

```
TCTATGTTTTTAGCCCTTGTTTTTTTCTATAATACATTGGATAGAGGAATGGAAAATAATAGAGCAGTA
TTACAAGGTAGGAATAAAAATTCATGAATAAAGTAAAAGTTCATACAATCAGTTAATTGGTAAAAGGTA
TTTACCAGTAGCGGAGCTACCTTTGTTCCAGGGGGTTCATCCCAAACCTCCTTCGACGAAAAATAATAT
TATTTTTATATTATTTTTACATGATTAAAAATATTTTTTATTCATATATAGTAGATGTTGAACCCTCTT
TAATTAGTTCGTATATTTAATTCTCGACCCTTCAATGAAAAATCCGACTTCATCACTGATATTTATTAT
ATTAATATTAAAAATAAATAAGGGGTAAATTGAATAAGAGTCGTCCCTTTAAGTTTAAGGACCGATTCA
ATAATAGTTCAATTAGATACGACTACGTCGAATACTGTAAAGTCTTTATAGGTCATTTGTCCATGAAAA
                              ERE(+)  HSE(-)              box III(+)
ATAATTTTTTGGAGTTAGAGTTGAAGTAAAATTTGAAATTTATTTATTTTTATTTTTTAAAAAATAATT
                                        box III(+)
TTCACTAAAGTAAACACTAATTTTTATGATCAAATACTATTTATTTACACTTTTTTGGCTATAGCCGAA
TAATTTTTGGGAAAAGAGTTAAAAATATCCCTCTACTATGATAAATTGATTAAATTTGCCCCCCGTTAT
       W box(-)
ACTATAGGGTCAAAAATACCCTTGTTGTTAGCAAAATTATCAAAAATATCCCTCCTTTTAACACTTGGT
GAGAGCCCACTTGGCATGACACTTGGCNNNNNNNNNNNNNNNTTTTTTTTCTTTTAATTTTTTCACTTCA
AATTTTATTAATCATTTATTTGTTGAAAAAGAAACTTATAATATTAAAAAAATTAATGAAGAACAATAT
                                            HSE(-)   W box(+)
TTTTCAGCCATTGGTGTCACTTCTCACCACCATTAATCCGATGGAAATTTTGTTTGACTAGAGTGGATT
                                    TCA-element (-)
AACAATTGATAATTGAAAGATGATTCTTGATTGAGGAAAAAGATGGTGTTTGGAAATAGGAAAATGGTT
GATTACTACTTTGATACATTGATATCTAACTAAACCATGGAACAACTTTCGCCACCATCATCGTTAATG
TCTACCATCTGGTTTTGGCGTCGAAGCCATTAACNCTTCAAGCTCGTCCACCATCAGTTCCTTTGTCAA
AGAAATGGAGAAAGGTCGTGGTTTTTGGTGTATTAGTGGTGGTGTAACCTGATATCGGTAACCGGAAAA
AACATATTTTTCATTTAATATTTTCTTAGTATTATTAGTTATCTTTTCAACAAAATAAATGATTAAGAA
                                              ABRE(-)
ATTTGAAGTGAAAAATTAAAAGAAAAAAAATATGATGTTGTGTCAAGCCACGTGGGCTCTCACTTTGTC
                                                ABRE(+)
                TCA-element (-)
ATGGTGGAGAAGTGTTAAAAAGATGAGTATTTTTAATAGCATTGCTAATCACAAGAGCACATTTCATCT
TATAGTATCACGAGGGACAAATTTAACTAATTTATTAAAATAGACAAGTATTTTTCACTGTTTCATAAT
TTTTTTGGGAAAAGGTGAAAAAAAATTGATGGCCAAATGCCTACTTAATTTAATTACAATTAAAGGAAG
ACTTGAGCTGTATATAAGTCTTTTAATATTCCAATAATCATCTTTTAAAAATCCCCTCCAATAATCACC
TTTCCCTCTCTGCATCTAGAAACTTCTTCGCTTCTCGTCTCATCTCATCTCATCCATG
```

Fig. S8. Nucleotide sequences of 5-flanking promoter regions and putative *cis*-acting elements of the *SGT1* . CCAAT-box, MYBHv1 binding site; GARE-motif, gibberellin-responsive element; HSE, *cis*-acting element involved in heat stress responsiveness; MBS, MYB binding site involved in drought-inducibility; TC-rich repeats, *cis*-acting element involved in defense and stress responsiveness; W box; transcription factor WRKY binding site; WUN-motif, wound-responsive element.

SRC2-1 promoter

```
AAAGGCATTTTAGTGTATAGTTTTGGACTTTCATACATGAGTGAATTTACAGTAGAAGTT
ACATACTTGATCAAATCCACGAATCTGATCGAATTGAGCAATTTTAGATCAAATCTATTT
       W box(-)
ATGAAAAGTGGTCAAGAAATATTGGAGTTGGTGATTTTATGACTCATTCCGTTTTTTAGT
TGTATAACTCATCTCATATAATATCTTTACGACAAAAGAGTCTTTATTCTTGGAATGGTT
                                     WUN-motif(+)
AATTCTTTTTTATATATAGTATACTAGTTATATAATTTCTAGGAAATTTCATCACAAGAT
                GARE-motif (-)
CGTAGATTATTCCATAAGGGTCTGTTTGGCAGTTTTAGAGCATATTCTGAAAATCTGCTT
GCACACGCTGTATACACGACGTTGGTTCGGAATTTGGCGCTTAATATTATAAGGAAAAAA
            GARE-motif (-)                   CCAAT-box(+)
AAGGATAAATACTATTCTGTTTGGATAACGCGTTTACTATTTTTATTACAACGGGTCTCT
AGCTTTTTCTGGAAATTAAATTAAATAACACATCAACAAAAGCCAAAAAGGGCCCCTTTA
                                MBS (+)
ATTTCCACAAAGTTTTGTACCTATCCAAATGAACGGTCATAAAAATAGAGCATCTTTATT
                      W box (+)
CTTACTTCGTCCGTTTTGCTATACTTGACTTTTTTTCTACAAAAATTTATTTTAATGTAA
TTATATTTATTTATAATAAAAAAAATATTTTATTATATTATTTTAACATTATTTTTATTA
TTAAATAATTAAATAACATGCATATAAATAAATTTATATTTTTAAAATATAATTAATAAA
ATTAATTAAATAAATAAAAATTAATAATTTTTTTAAGTAGAATATTAAATTAATAAAAAT
CAATTATTTTAAAATAAAATAAATATAATTATTTGTACATTTTAATTTATAAATAATGTG
TACAAATATATTATTTATATATAACATCAAAAATATATTATTTATACTACACATCTTTTC
               TC-rich repeats (-)
TTAGTAAGAGCAGGAATATATTGTAGAAAATAACGGCACGAAAAAGCGACCCCATAATTG
                                            TC-rich repeats (+)
ATAGTGTATGCTTACTGTTACTGACCTATTCCTCCGTCTCATTTTCCGTGATATATTTTC
TTGAGACACAAAATTTTAGTTATAAAGAAAGACTTAATTATATTTGTTAAATTAATTTTT
ATATTAAAAAAATATATTGTTATTATTTTTAGTAAAGTGGAATTAATAAAAATTTAAAAA
ATAATTATGTTAGTATTAAAAAGTTACCAAATCAAAAAATATTAGCAAATAAAATGGGAG
CAATTCAACTTTACTGCTACCTTAGAGTCGGTTTATTCGAAAGTTCCTGTTTATTTCCTT
TCGTTATTATACCCTTCCCTTATAAATTCCTCCAATTCCCCCATCAACCCAAACCAACAC
      HSE(+)                          MBS(-)      WUN-motif(-)
GCACAAAAATTTTCAGAGTTTCTCTTTCAACTTCCAGTTACAATTTTCATCCTCCAAATT
TCCTTCTGAATCTCTTTGCAATG
```

Fig. S9. Nucleotide sequences of 5-flanking promoter regions and putative *cis*-acting elements of the *SRC2-1* . CCAAT-box, MYBHv1 binding site; GARE-motif, gibberellin-responsive element; HSE, *cis*-acting element involved in heat stress responsiveness; MBS, MYB binding site involved in drought-inducibility; TC-rich repeats, *cis*-acting element involved in defense and stress responsiveness; W box; transcription factor WRKY binding site; WUN-motif, wound-responsive element.

Supplementary Tables

Supplementary Table S1. Primers used in the qPCR and the validation of qPCR

Gene	Accession no.	Forward primers	Reverse primers	Length of PCR product	Specificity screen NCBI	Peak number of Melting curve	Cq of the no-template controls	slope	PCR efficiency(%)	y intercept	R^2
SGT1	JN252483	5'-GCTGATGAGGATGCAAGAAGAGC-3'	5'-CCAAGAATGAAGAGAAAAAAGGA-3'	213	specific product	1	N/A	-3.401	0.968027521	33.453	0.990
SRC2-1	DQ465394.1	5-ATGTCTTCTTATGCTGATGG-3	5-AGTCAGAAACTATTATAAT-3	214	specific product	1	N/A	-3.247	1.03224748	30.737	0.995
SAR82A	AF313766	5'-CAGGGAGATGAATTCTGAGGC-3'	5'-CATATGAACCTCTATGGATTTCTG-3'	165	specific product	1	N/A	-3.421	0.960253281	36.976	0.997
HIR1	AY529867	5-GACATGGTCCTGGTAACCCA-3	5-CCCAACAGAAGCCTGAAGAA-3	150	specific product	1	N/A	-3.321	1.000387455	32.819	0.961
DEF1	AF442388	5'-CAAGGGAGTATGTGCTAGTGAGAC-3'	5'-TGCACAGCACTATCATTGCATAC-3'	267	specific product	1	N/A	-3.423	0.959482533	30.912	0.993
BPR1	AF053343	5-CAGGATGCAACACTCTGGTGG-3	5-ATCAAAGGCCGGTTGGTC-3	310	specific product	1	N/A	-3.346	0.9900515	32.432	0.993
PR4	AF244122.1	5-CAACCCGCAGAACATCAACTGG-3	5-CCTCAAGCATCTACCGCAAGCA-3	160	specific product	1	N/A	-3.363	0.983140817	36.584	0.992
PO2	DQ489711	5-TGATTGCTTTGTTCAGGGTT-3	5-ATGATGGACCTCCAACGAGA-3	224	specific product	1	N/A	-3.315	1.002899348	35.884	0.999
CaACTIN	GQ339766(NCBI)	5'-CCTCTCAACCCTAAGGCCAACAG-3'	5'-ACGTCCAGCAAGATCCAAACGAA-3'	225	specific product	1	N/A	-3.321	1.000387455	35.521	0.995
18S ribosomal RNA	EF564281(NCBI)	5'-CCGGTCCGCCTATGGTGTGCACCGGTCGTC-3'	5'-GCAGTTGTTCGTCTTTCATAAATCCAAGAA-3'	285	specific product	1	N/A	-3.320	1.000805255	35.632	0.993

SupplementaryTable S2. Oligonucleotides for plasmid constructs used in this study

Analysis	Gene	Sequence of the oligonucleotide
BiFC/*E.coli* overexpression	SRC2-1 F	5'-GGGGACAAGTTTGTACAAAAAAGCAGGCTTCATGGAGTTACGTCCGTTAGA-3'
	SRC2-1 R	5'-GGGGACCACTTTGTACAAGAAAGCTGGGTCCTAAAAATCAAACCCACCACC-3'
	SGT1 F	5'-GGGGACAAGTTTGTACAAAAAAGCAGGCTTCATGGCGTCCGATCTCTCAAC-3'
	SGT1 R	5'-GGGGACCACTTTGTACAAGAAAGCTGGGTCCTATAGTATTCCCATTGCTT-3'
VIGS	SGT1 240 F	5'-GGGGACAAGTTTGTACAAAAAAGCAGGCTTCTCAAGATTCACTAGCTTAAT-3'
	SGT1 240 R	5'-GGGGACCACTTTGTACAAGAAAGCTGGGTCCGTCTGGTTTCTGATAGAAT-3'
	SRC2-1 275 F	5'-GGGGACAAGTTTGTACAAAAAAGCAGGCTTCCAGCGCCTGAGAAGAAGAAAGAC-3'
	SRC2-1 275 R	5'-GGGGACCACTTTGTACAAGAAAGCTGGGTCCCCTAATCCACCTCCTCCGAACT-3'
Subcellular localization	NLS	5'-GGCCCTAAAAAGAAGCGTAAGGTT-3'
	nls	5'-GGCCCTAAAAACAAGCGTAAGGTT-3'

Supplementary Table S3. *Cis*-acting element found in the promoter of SGT1 and SRC2-1

Gene	ABRE	Box III	CCAAT-box	ERE	GARE-motif	HSE	MBS	TCA-element	TC-rich repeats	W box	WUN-motif
SGT1	2	2	0	1	0	2	0	2	0	2	0
SRC2-1	0	0	1	0	2	1	2	0	2	2	2

ABRE, cis-acting element involved in the abscisic acid responsiveness; Box III, protein binding site; CCAAT-box, MYBHv1 binding site; ERE, ethylene-responsive element;GARE-motif, gibberellin-responsive element; HSE, cis-acting element involved in heat stress responsiveness; MBS, MYB binding site involved in drought-inducibility;TC-rich repeats, cis-acting element involved in defense and stress responsiveness;TCA-element, cis-acting element involved in salicylic acid responsiveness; W box; transcription factor WRKY binding site;WUN-motif, wound-responsive element
